# Ψ-co-mAFiA: concurrent detection of pseudouridine and m^6^A in single RNA molecules

**DOI:** 10.1093/bioinformatics/btaf536

**Published:** 2025-09-26

**Authors:** Adrian Chan, Isabel S Naarmann-de Vries, Christoph Dieterich

**Affiliations:** Bioinformatics and Systems Cardiology, Klaus Tschira Institute for Integrative Computational Cardiology, Im Neuenheimer Feld 669, 69120 Heidelberg, Germany; Department of Internal Medicine III, University Hospital Heidelberg, Im Neuenheimer Feld 410, 69120 Heidelberg, Germany; Bioinformatics and Systems Cardiology, Klaus Tschira Institute for Integrative Computational Cardiology, Im Neuenheimer Feld 669, 69120 Heidelberg, Germany; Department of Internal Medicine III, University Hospital Heidelberg, Im Neuenheimer Feld 410, 69120 Heidelberg, Germany; German Center for Cardiovascular Research (DZHK) — Partner Site Heidelberg/Mannheim, Im Neuenheimer Feld 669, 69120 Heidelberg, Germany; Bioinformatics and Systems Cardiology, Klaus Tschira Institute for Integrative Computational Cardiology, Im Neuenheimer Feld 669, 69120 Heidelberg, Germany; Department of Internal Medicine III, University Hospital Heidelberg, Im Neuenheimer Feld 410, 69120 Heidelberg, Germany; German Center for Cardiovascular Research (DZHK) — Partner Site Heidelberg/Mannheim, Im Neuenheimer Feld 669, 69120 Heidelberg, Germany

## Abstract

**Summary:**

The development of third-generation sequencing technologies enables the detection of RNA modifications at single-molecule resolution. Specifically for direct RNA sequencing on the ONT platform, we have previously developed an m^6^A detection algorithm called mAFiA. Here, we present the updated method, now covering all 18 DRACH m^6^A contexts as well as the identification of pseudouridine sites (Ψ). Our modification level predictions compare favorably with orthogonal methods and respond to knockdown or knock out of writer proteins. The simultaneous detection of multiple modifications on a single RNA molecule opens up the possibility to study cross-modification interactions.

**Availability and implementation:**

Ψ-co-mAFiA is available at https://github.com/dieterich-lab/psi-co-mAFiA and licensed under GPLv3.0. An archived version of the software is available on Zenodo at https://doi.org/10.5281/zenodo.16797676.

## 1 Introduction

So far, more than 170 chemical RNA modifications have been identified (Delaunay *et al.* 2024). Among them, N6-methyladenosine (m^6^A) is the single best characterized RNA modification. Up to 1.0% of all adenosine residues in mRNAs is m^6^A-modified (Jones *et al.* 2020) and these dynamic modifications have been attributed to changes in RNA splicing, translation and stability. Other important modifications such as pseudouridine (Ψ) occur at a lower level of 0.2%–0.6% of uridine residues in mRNAs (Jones *et al.* 2020). Emerging evidence shows that pseudouridylation in mRNA can modulate translation and suppress nonsense-mediated decay, potentially affecting protein synthesis during stress or development (Chen *et al.* 2024).

Although levels of m^6^A and Ψ can each be estimated through various chemical assays (Dai *et al.* 2023, Liu *et al.* 2023, Zhang *et al.* 2023), simultaneous measurement of multiple modifications has not been possible until recently, hindering the study of the interplay of different RNA modifications types. Nanopore sequencing encodes modified base signals in each single RNA molecule, and has emerged as an enabling technology for the study of the epitranscriptome (Leger *et al.* 2021, Wan *et al.* 2022). For m^6^A specifically, a multiple-instance learning approach has been developed for biological mRNAs that does not require single-molecule training labels (Hendra *et al.* 2022). In our previous work (Chan *et al.* 2024), we demonstrated the feasibility of training an m^6^A detection algorithm using synthetic mRNAs that mirror sequences with modification sites on the human transcriptome. Here, we report on an extended algorithm, Ψ-co-mAFiA, which covers all 18 m^6^A consensus motifs, known as DRACH motifs (D = A/G/U; R = A/G, H = A/C/U) as well as 16 high-frequency sequence contexts of Ψ in mammals (see Table 1, available as supplementary data at *Bioinformatics* online and Supplementary Methods). The simultaneous quantification of both modifications on single RNA molecules opens up new opportunities for research.

## 2 Results

### 2.1 Algorithm

**Figure 1.**
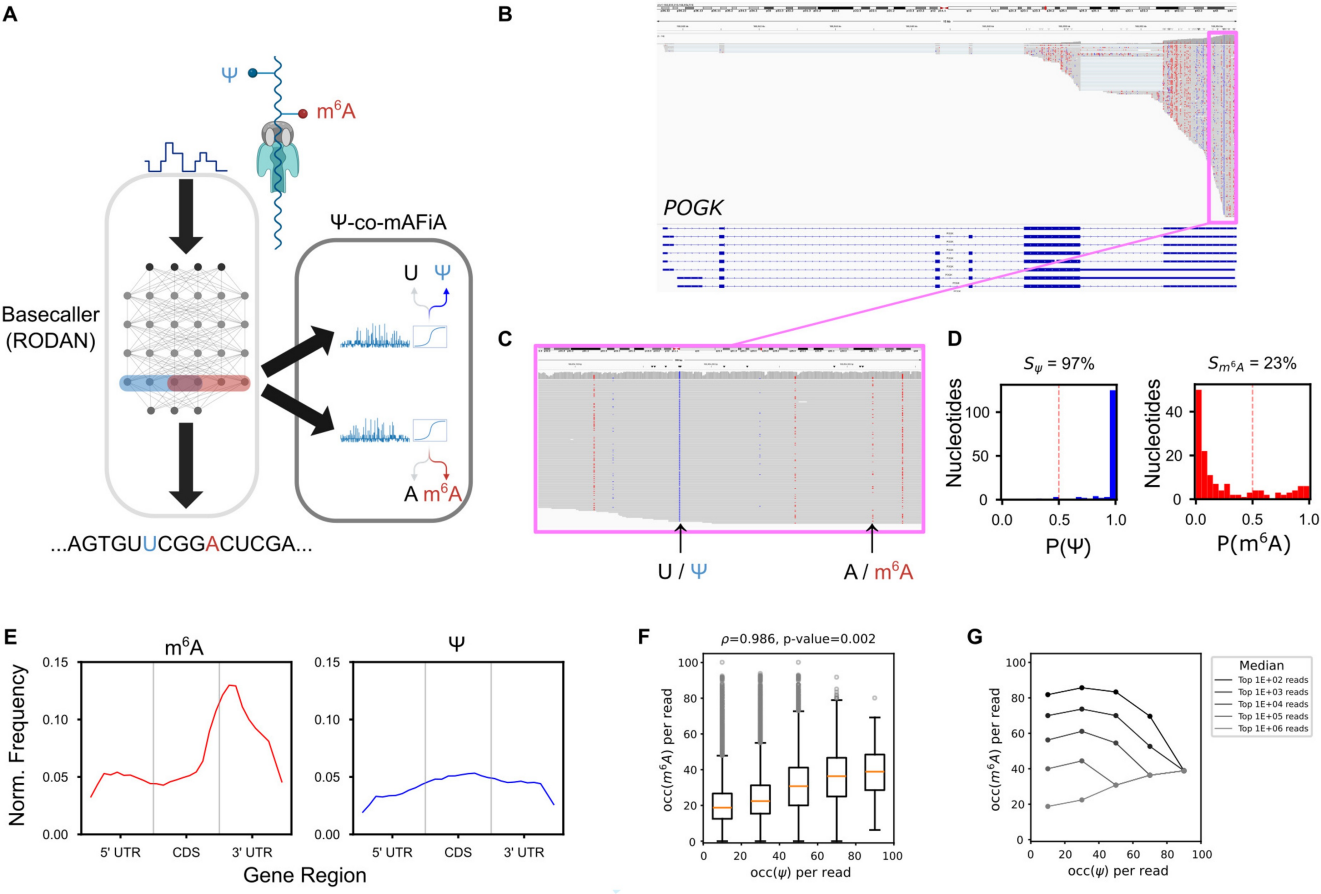
(A) Schematic representation of Ψ-co-mAFiA. A basecaller RODAN (Neumann *et al.* 2022) (left box) receives raw data from direct RNA sequencing and interprets the signal as a string of canonical RNA bases A, C, G, U. Corresponding to specific locations of A and U in the molecule, Ψ-co-mAFiA (right box) extracts internal features from the basecaller and predicts the probability that the nucleotide is modified (U to Ψ, A to m^6^A). Created with BioRender.com. (B) IGV snapshot of full-length epitranscriptomic map of the gene *POGK* expressed in HEK293 WT. Each row corresponds to a single mRNA molecule. Blue = Ψ; red = m^6^A. Annotated isoforms are shown at the bottom. (C) Zoom-in of the highlighted exon (pink box) in panel B, showing both highly and lowly Ψ and m^6^A modified sites. (D) Distribution of single-nucleotide modification probability P(Ψ) and P(m^6^A) at two different sites indicated by arrows in panel C. The site-level stoichiometry S (shown above the histogram) is calculated by counting the fraction of nucleotides aligned to the site with modification probability above 0.5. (E) Metatranscript profile of m^6^A (red) and Ψ (blue) modifications on HEK293 wildtype mRNA. (F) m^6^A–Ψ interaction at single-molecule resolution in HEK293 WT. The modification occupancy per molecule, occ, is defined as the number of sites on a single read with modification probability over 0.5, normalized by the total number of potentially modified locations on the same read (i.e. that fall into our trained motifs). Boxplot showing distribution of modification occupancy per molecule occ(m^6^A) as a function of occ(Ψ), binned in intervals of 20%. Orange lines mark the median of each distribution. The overall levels of the two modifications in a molecule are positively correlated (Pearson correlation ρ=0.986, *P*-value = 0.002). Number of reads in each bin (from lowest to highest): 825126, 806033, 151412, 12498, 427. (G) Median of top 1E+X (X = 2 to 6) reads occ(m^6^A) in each interval of panel F. The top 100 to 10 000 outliers reveal a slight anti-correlation between the two modifications.

As described in our previous work (Chan *et al.* 2024), our RNA modification detection algorithm is based on the extraction of hidden features from a fully convolutional neural network (Neumann *et al.* 2022) during the direct RNA sequencing base call stage (Fig. 1A). Briefly, we build classifiers for every motif occurrence based on these hidden features (see Supplementary Methods and Figs 1 and 2, available as supplementary data at *Bioinformatics* online for details on training and testing). Figure 1B shows the entire *POGK* gene locus as an example. This gene is expressed in the human cell line HEK293 and covered by Nanopore long direct RNA sequencing reads, which start from the 3′ end. Ψ-co-mAFiA is then used to predict the modification probabilities of single nucleotides in each RNA molecule. The modification stoichiometry (denoted *S* thereafter) of a specific site is given by the fraction of nucleotides aligned to the same genomic position that carry a read-level modification probability above 50% (Chan *et al.* 2024) (Fig. 1C for a close up and panel D for estimated stoichiometries of 2 selected sites). In HEK293 mRNA, the distribution of highly modified sites (S≥50%) along a meta-transcript shows that m^6^A is concentrated at the 3′ UTR, while Ψ is evenly distributed throughout the gene body (Fig. 1E).

### 2.2 Validation

To validate the numerical precision of our method, we have compared single site stoichiometries in the HEK293 cell line with orthogonal measurements from chemical assays (Dai *et al.* 2023, Liu *et al.* 2023, Zhang *et al.* 2023). The correlation of our predictions with published values is >90% on both m^6^A and Ψ (Fig. 3, available as supplementary data at *Bioinformatics* online). Further experiments in HEK293 cells using either depletion of writer proteins (*METTL3* and *TRUB1*, Fig. 4, available as supplementary data at *Bioinformatics* online) or *in vitro* transcription (IVT) samples (Fig. 5, available as supplementary data at *Bioinformatics* online) show that our approach accurately predicts quantitative changes on individual modification sites. All site-level results are made available through the Sci-Modom database (Boileau *et al.* 2025) under SMID WrBiNJCZ.

### 2.3 m^6^A-Ψ interaction at single-molecule resolution

A previous study (Huang *et al.* 2024) reported an anti-correlation of aggregate levels of m^6^A and Ψ in certain transcripts. However, the interplay between the two modifications within single RNA molecules remained unexplored. Taking only the 18 DRACH and 16 pseudouridine motifs into account, we define the occupancy occ(m^6^A/Ψ) as the number of sites on a single read with modification probability over 0.5, normalized by the total number of potentially modified locations on the same read. These read-level predictions of our algorithm reveal a startling picture: taking into account the full range of modification levels between 0 and 100%, the occurrence of m^6^A and Ψ in each molecule is, in fact, positively correlated (Fig. 1F). Within the subset of molecules with highly saturated modification levels (the top 100 to 10 000 modified reads) we see an anti-correlation between m^6^A and Ψ levels (Fig. 1G). The latter result agrees with the observation of Huang *et al.* (2024), but also highlights the insufficiency of methods based on counting highly modified sites alone. The interaction between the two RNA modifications is more complex than initially hypothesized and warrants further investigation.

## 3 Conclusion

In this work, we have demonstrated the possibility to simultaneously detect m^6^A and Ψ in a complex mammalian transcriptome. To this end, we use an established base calling algorithm, extract features from hidden layers and train a classifier for m^6^A and Ψ on realistic synthetic data. We have shown non-trivial interdependence between the two modification types at single-molecule resolution. Future work could include additional modifications that are synthesizable in the laboratory, as well as additional sequence contexts found in other non-mammalian species, which are not covered by our current method. As our approach uses fully supervised machine-learning techniques, its range of detection is limited by the amount of labeled training data that we can synthesize. More broadly for all methods based on third-generation sequencing, direct RNA signals from neighboring nucleotides interfere with one another, which means that multiple modifications in close proximity tend to distort the predictions. Such incidents are relatively rare in mRNAs, but common in rRNAs and tRNAs. We envisage that such issues can be overcome by generating training data that contains multiple modified species within a single molecule, which is technically feasible though out of the scope of our current study. As the overall accuracy of Nanopore sequencing continues to improve, we expect the technology to be an indispensable tool in the emerging field of epitranscriptomics.

## Author contributions

Adrian Chan (Conceptualization, Formal analysis, Investigation, Methodology, Software, Validation, Visualization, Writing—original draft), Isabel S. Naarmann-de Vries (Investigation, Visualization, Writing—original draft, Writing—review & editing), and Christoph Dieterich (Funding acquisition, Investigation, Project administration, Resources, Supervision, Writing—review & editing)

## Supplementary Material

btaf536_Supplementary_Data

## Data Availability

Source code and documentation are available under GPLv3.0 at https://github.com/dieterich-lab/psi-co-mAFiA. An archived version of the software, which was used to produce all results, is available under https://doi.org/10.5281/zenodo.16797676.
