# Supplementary material for: Ψ-co-mAFiA: concurrent detection of pseudouridine and m6A in single RNA molecules
Source: Bioinformatics. 2025 Sep 26;41(10):btaf536. doi: 10.1093/bioinformatics/btaf536 (PMC12552086; doi:10.1093/bioinformatics/btaf536)
Supplement: btaf536_Supplementary_Data [file btaf536_supplementary_data.zip › Supplementary_Materials_final.pdf]

Supplementary Data for
Ψ-co-mAFiA: Concurrent detection of pseudouridine and m^{6}A in single RNA molecules

Adrian Chan, Isabel S. Naarmann-de Vries, Christoph Dieterich

September 20, 2025

1 Supplementary Methods

1.1 RNA oligos

RNA oligo design: The 5-mer motifs were chosen based on their frequencies of appearance in a consensus of orthogonal measurements. For m^{6}A, GLORI (Liu *et al.*, 2023) and miCLiP (Boulias *et al.*, 2019; Koh *et al.*, 2019; Körtel *et al.*, 2021) were used, which lead to the DRACH motifs. For pseudouridine, the motifs were derived from BID-Seq (Dai *et al.*, 2023) and PRAISE (Zhang *et al.*, 2023). The full 21-mer sequences were determined as follows: first, for each 5-mer we collected a shortlist of candidate 21-mer sequences from the human transcriptome that have a high probability of carrying an m^{6}A or pseudouridine in the center. Then, we performed an iterative ranking procedure such that the ±8nt flanking sequence chosen for each 5-mer has maximal edit distance from the context of other 5-mers. These flanking sequences then serve as the barcodes that allow us to clearly distinguish between different 5-mers in the pool of oligonucleotides. This oligo design focuses on the most abundant motifs in mammals. Other motifs that are less frequent, but biologically significant are currently not covered. The design of our approach however allows to extend the training data set with additional motifs/ sequence context. For pseudouridine, PSU_M9 (UUUUU) was excluded, as the localization of the modification signal in consecutive Us is highly uncertain.

RNA oligo ligation and sequencing: The designed 21 nt-long RNA oligos (Table S1) were ordered with a 5′ phosphate in unmodified and modified form (either m^{6}A or Ψ in the central position) from GenScript and concatenated by Random Ligation as described previously (Chan *et al.*, 2024). Whenever low efficiency of Random Ligation was observed, alternative oligos with the same 5-mer were choosen from the list described above (_S0, _S1, etc.). Oligos were pooled as listed in Supplementary Table 2 and sequenced as described employing the SQK-RNA002 direct RNA-sequencing kit from Oxford Nanopore technologies (Chan *et al.*, 2024).

1.2 Data used in this study

The oligo training data generated for this study have been deposited in the European Nucleotide archive under accession number PRJEB82824 as listed in Supplementary Table 3. Training data for the original mAFiA publication (Chan *et al.*, 2024) and HEK293 data have been deposited in the European Nucleotide archive under accession number PRJEB74106. Furthermore, public METTL3-KO data (PRJEB40872) and METTL3-KD and TRUB1-KD data (PRJEB72637) have been used in this study as listed in Supplementary Table 2.

1.3 Training and Validation

Training and Validation was performed as described previously (Chan *et al.*, 2024). In brief, RNA oligo direct RNA-seq data are basecalled with RODAN. Reference oligo sequences are mapped locally to each read and then chained together to infer the ligated sequence in each RNA strand. Afterwards, each central A or U nucleotide in an oligo is classified into one of the target motifs. At the read location corresponding

to each of these A or U nucleotides, a 768-dimensional feature vector is extracted from the last convolution layer of RODAN. In case of sample imbalance between unmodified and modified classes, the more numerous class is randomly subsampled to match the size of the smaller class. For each motif, the collected feature samples are split into 0.75-0.25 train-validation sets. Fourfold cross-validation was performed to control for overfitting, and the maximum fluctuation in AUPRC between the folds is below 1% for each motif. The test data set is derived from HEK293 cells, which is not balanced and the modification ratio can range from 0% to 100%. The variation between replicates was assessed using two HEK293 WT data sets. We find an RMS difference on the prediction per site of 8.5%.

1.4 Analysis of m^6A-Ψ interaction at single-molecule resolution

Taking only the 18 DRACH and 16 pseudouridine motifs from our model into account, we define the occupancy $occ(m^6A/\Psi)$ as the number of sites on a single read with modification probability over 0.5, normalized by the total number of potentially modified locations on the same read. The Pearson's rho and p-value are given in Figure 1F. The p-value is calculated from the median value of the m^6A distribution in each bin and comparing it with the bin center values (10%, 30%, 50%, 70%, 90%). The null-model assumes that these two series of median values are randomly distributed with respect to each other.

1.5 Software accession and hardware architecture

Source code and documentation for Ψ-co-mAFiA are available under GPLv3.0 at `https://github.com/dieterich-lab/psi-co-mAFiA`. We have analyzed one chromosome per job. Each job uses 4 CPUs, 64 GB memory, 1 NVIDIA Quadro RTX 6000.

An exemplary output BED file (genomic coordinates) is provided as Supplementary Material - HEK293_psi_co_mafia_predictions.bed.gz .

Figure S1

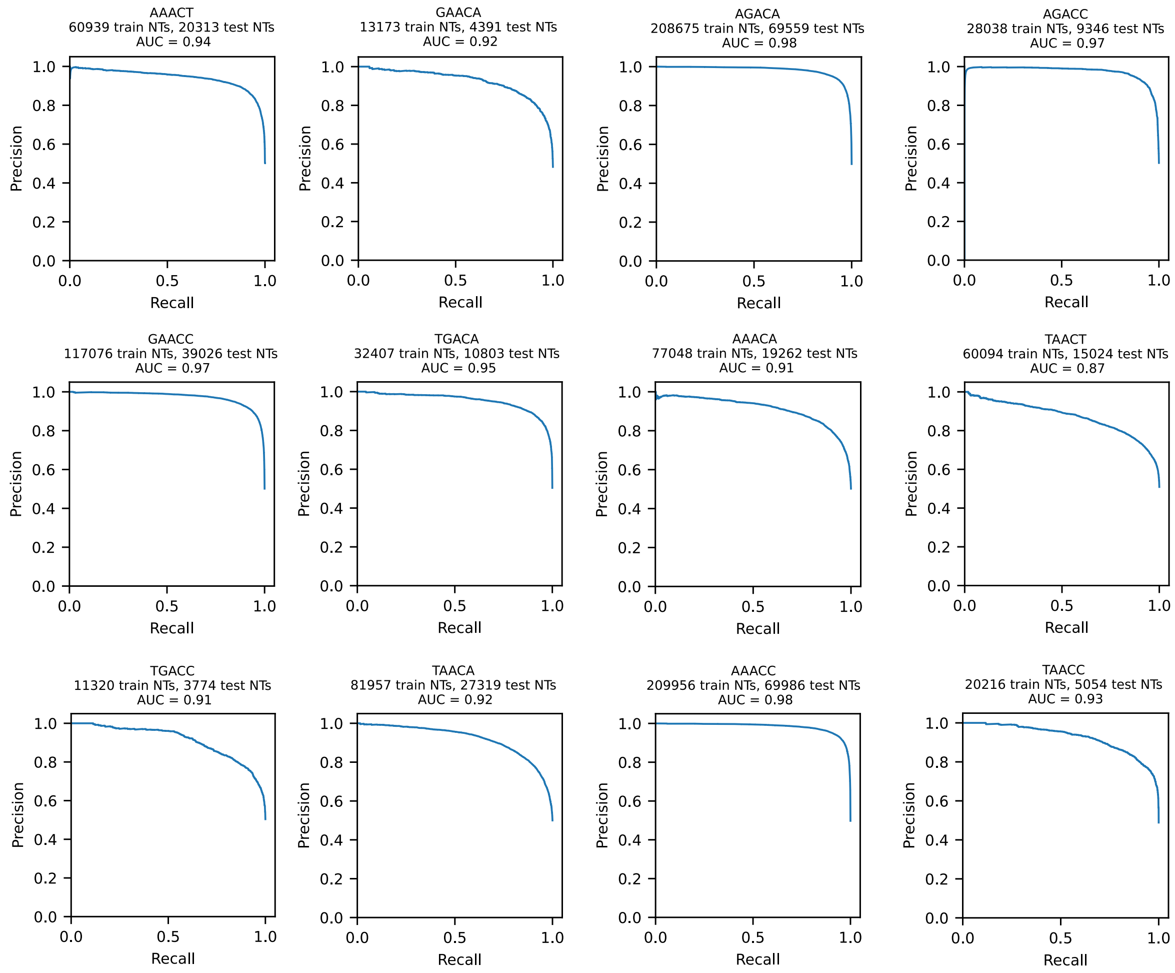

Figure 1: Model validation - Ψ-co-mAFiA models trained on 75% of the synthetic RNA oligo data set and validated on 25% of the data set. Precision-recall curves (PRCs) calculated from the $P(m^6A)$ distributions, with area-under-curve (AUC) given in the legend for the 12 new DRACH motifs, which were not part of (Chan *et al.*, 2024).

Figure S2

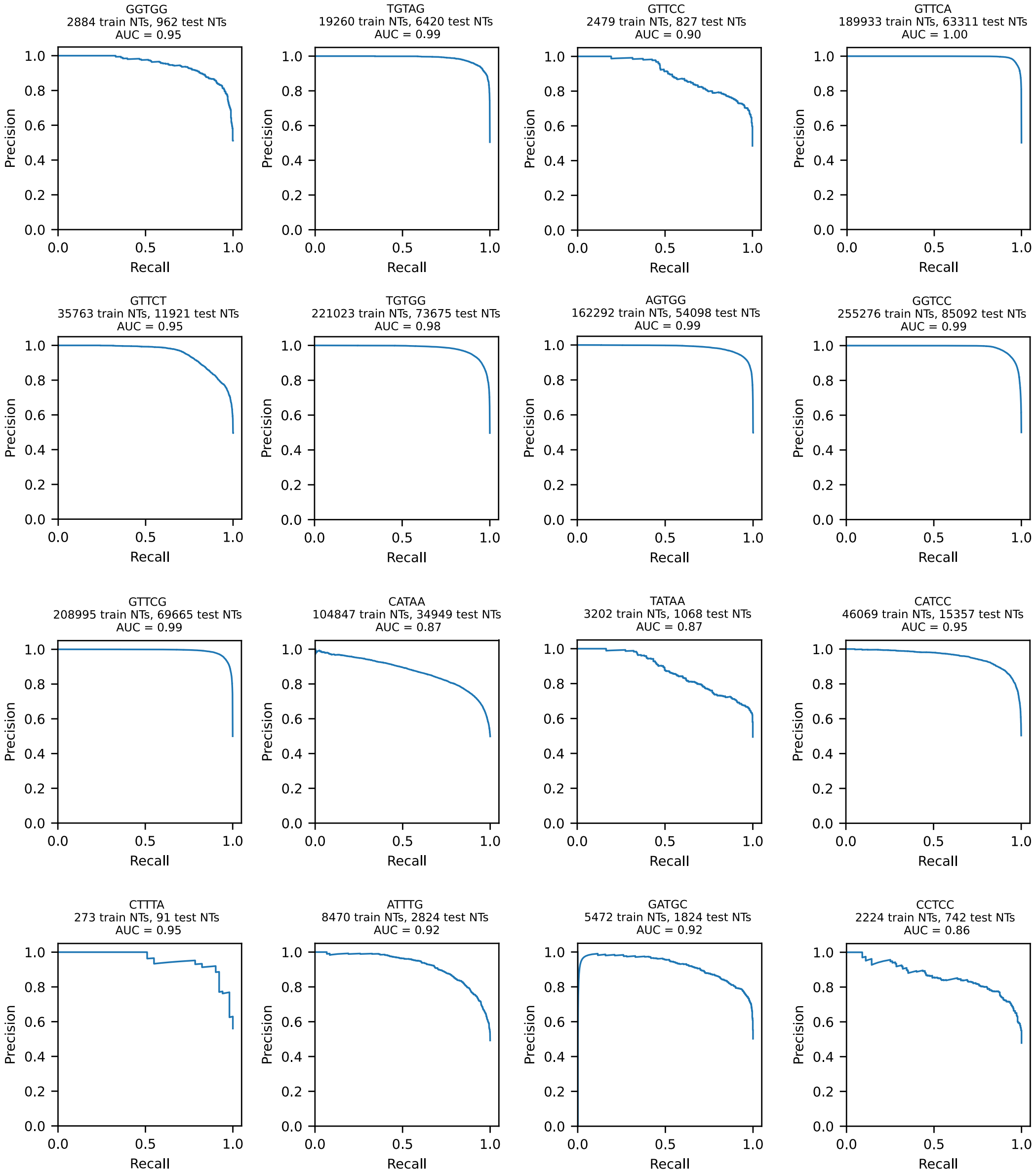

Figure 2: Model validation - Ψ-co-mAFiA models trained on 75% of the synthetic RNA oligo data set and validated on 25% of the data set. PRCs calculated from the $P(\Psi)$ distributions, with AUC given in the legend for the 16 pseudouridine motifs.

Figure S3

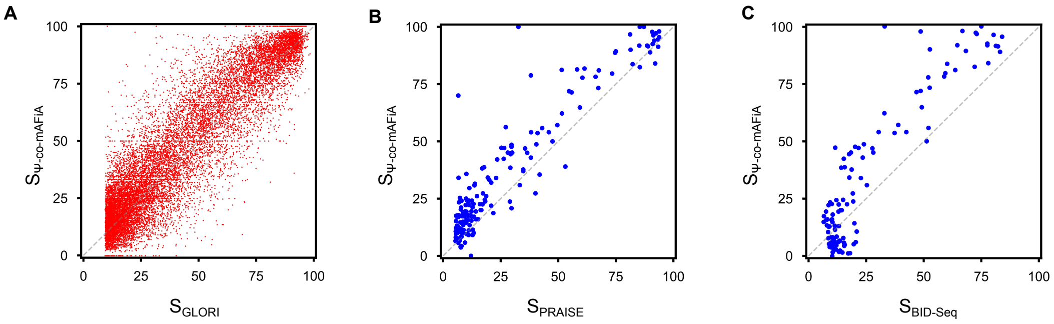

Figure 3: Validation of Ψ-co-mAFiA with orthogonal measurements. Site-level data (x-axes) from Liu *et al.* (2023), Zhang *et al.* (2023) and Dai *et al.* (2023). HEK293 WT nanopore data (y-axis) from (Chan *et al.*, 2024).

A: Comparison of site-level m^6A stoichiometry predicted by Ψ-co-mAFiA (y-axis) in HEK293 WT mRNA to the measurements of GLORI (x-axis) (Liu *et al.*, 2023) Minimum site coverage 20. N=15928. Correlation 0.92. GLORI does not report values of S below 10%

B: Comparison of site-level Ψ stoichiometry predicted by Ψ-co-mAFiA (y-axis) in HEK293 WT mRNA to the measurements of PRAISE (x-axis) (Zhang *et al.*, 2023) N=180. Correlation 0.94. PRAISE does not report values of S below 5%

C: Comparison of site-level Ψ stoichiometry predicted by Ψ-co-mAFiA (y-axis) in HEK293 WT mRNA to the deletion ratios measured by BID-Seq (x-axis) (Dai *et al.*, 2023) N=115. Correlation 0.92. BID-Seq does not report values of S below 10%

Figure S4

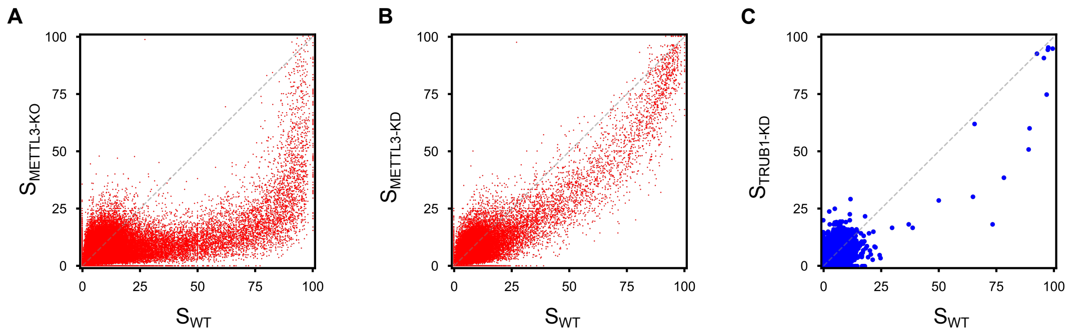

Figure 4: Assessment of Ψ-co-mAFiA performance employing writer knock-out and knock-down experiments. Raw Nanopore data from Hendra *et al.* (2022) (PRJEB40872) and Huang *et al.* (2024) (PRJEB72637).

A: Comparison of site-level m^6A stoichiometry predicted by Ψ-co-mAFiA in HEK293 METTL3 knock-out (METTL3-KO, y-axis) versus wild-type (WT, x-axis). N=76090.

B: Comparison of site-level m^6A stoichiometry predicted by Ψ-co-mAFiA in HEK293 METTL3 knock-down (METTL3-KD, y-axis) versus wild-type (WT, x-axis). N=34635.

C: Comparison of site-level Ψ stoichiometry of GUΨCN motifs (Dai *et al.*, 2023) predicted by Ψ-co-mAFiA in HEK293 TRUB1 knock-down (TRUB1-KD, y-axis) versus wild-type (WT, x-axis). N=4368.

Figure S5

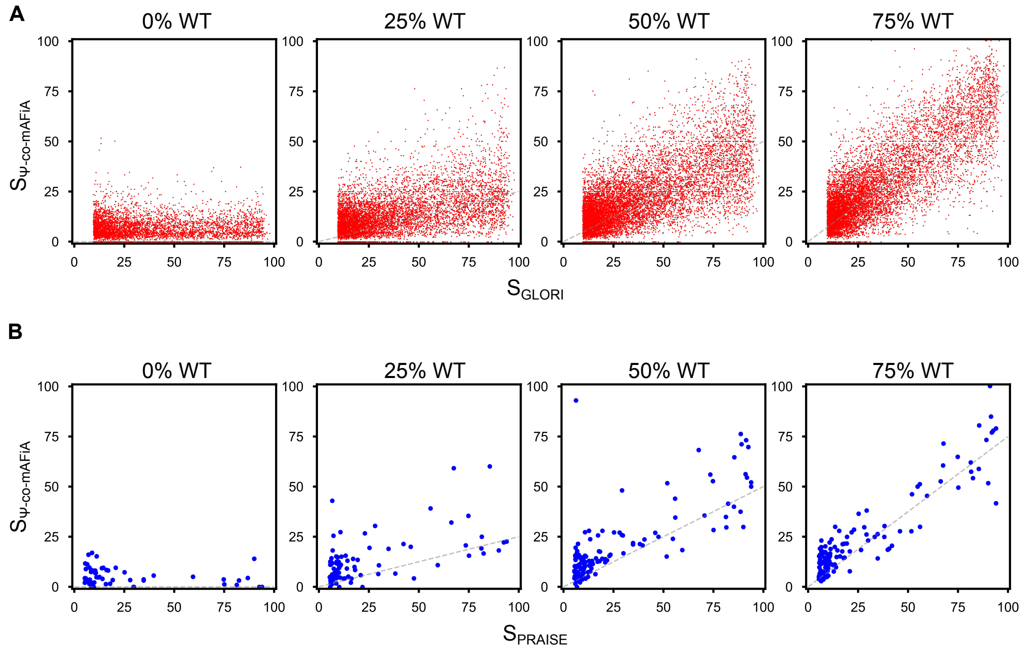

Figure 5: IVT-WT mixing experiments. Data from (Chan *et al.*, 2024) (PRJEB74106).

A: m^6A stoichiometry predicted by Ψ-co-mAFiA in HEK293 IVT-WT mixtures (y-axis) versus GLORI (x-axis), at 4 different concentrations of WT (0%, 25%, 50%, 75%). Gray dashed lines indicate slopes of 0, 0.25, 0.5, 0.75 respectively. N=5111, 7643, 10691, 9885.

B: Ψ stoichiometry predicted by Ψ-co-mAFiA in HEK293 IVT-WT mixtures (y-axis) versus PRAISE (x-axis). N=60, 87, 126, 126.

Table S1

RNA oligo name	Motif	Sequence
M6A_M6_S0	AAACU	ACAAAUGAAAA/m6ACUCUGGGCUG
M6A_M7_S0	GAACA	UCUUAUUGGAA/m6ACAACCACUGA
M6A_M8_S0	AGACA	CUGGUGAGAGA/m6ACAUGAACACU
M6A_M9_S0	AGACC	CUGCGGCAAGA/m6ACCUUCAAUCG
M6A_M10_S0	GAACC	AAUCGCUUGAA/m6ACCCAGGAGGU
M6A_M11_S0	UGACA	UCUCCAGCUGA/m6ACAGCAGAAAA
M6A_M12_S0	AAACA	AAACAAACAAA/m6ACAAACAGCCU
M6A_M12_S1	AAACA	AUUCCACCAAA/m6ACAUGGAGGAG
M6A_M13_S0	UAACU	CUAUUUGGUAA/m6ACUGGAGUAAC
M6A_M13_S1	UAACU	CACUUUAAUAA/m6ACUGUUGCUUA
M6A_M14_S0	UGACC	GUGACCCGUGA/m6ACCCAUGACCC
M6A_M15_S0	UAACA	UUGCCAUGUAA/m6ACAAAUUACUG
M6A_M16_S0	AAACC	GAAUUGACAAA/m6ACCCAUCUUGA
M6A_M17_S0	UAACC	AUUUAUCAUAA/m6ACCAUGUGUAA
M6A_M17_S1	UAACC	CAUAAUUGUAA/m6ACCAAUUUCAC
PSU_M0_S0	GGUGG	UUUGGCGUGGU/psUGGUGAGUGAG
PSU_M1_S0	UGUAG	CUUGAUGUUGU/psUAGAUGAGUUG
PSU_M1_S1	UGUAG	GAGUAAGAUGU/psUAGGAUUAUAA
PSU_M2_S0	GUUCC	ACACCUGGGUU/psUCCAAUCCCAG
PSU_M2_S1	GUUCC	UAAUGUUGGUU/psUCCAGUCCUUC
PSU_M3_S0	GUUCA	GGAAAACAGUU/psUCAACUCUGAC
PSU_M4_S0	GUUCU	AGGCCUAGGUU/psUCUAGUCCUAG
PSU_M4_S1	GUUCU	UUCAGCAUGUU/psUCUAACCAUGC
PSU_M4_S2	GUUCU	AGCCUGGAGUU/psUCUAAUCUUCA
PSU_M5_S0	UGUGG	GUUUGAGAUGU/psUGGGCUUCAAU
PSU_M6_S0	AGUGG	CGUCGAGCAGU/psUGGUUCGGCUC
PSU_M6_S1	AGUGG	ACCCGGGAAGU/psUGGAGGUUGCA
PSU_M7_S0	GGUCC	AGCCCCGAGGU/psUCCGAGGUCCG
PSU_M8_S0	GUUCG	CAGGGCUGGUU/psUCGAAUCCAUU
PSU_M9_S0	*UUUUU*	*AUGCAAUCUUU/psUUUUUUUUUUA*
PSU_M9_S1	*UUUUU*	*UCAGGAAAUUU/psUUUGGAUGGUA*
PSU_M10_S0	CAUAA	UUCGAAAACAU/psUAAAGAAAAGA
PSU_M11_S0	UAUAA	AAGCCAUGUAU/psUAAAUGGCUUC
PSU_M12_S0	CAUCC	AAGGAGGGCAU/psUCCCCCCUGAC
PSU_M13_S0	CUUUA	CACAGCAGCUU/psUUAGCGCUGGU
PSU_M14_S0	AUUUG	GAUAAAGGAUU/psUUGUAGUUGAA
PSU_M15_S0	GAUGC	AGAAGAUAGAU/psUGCUUCUAAGU
PSU_M16_S0	CCUCC	CCUCCAUCCCU/psUCCAGGAUGGG

Table 1: Oligo sequences used for training Ψ-co-mAFiA

2 Table S2

sequencing run name	Description	Usage	ENA Project/ Sample
HEK293 WT	HEK293 WT mRNA	Figure 1, S3, S4, S5	PRJEB74106/ SAMEA115428103
0% WT	HEK293 IVT	Figure S5	PRJEB74106/ SAMEA115526579
25% WT	mix of HEK293 WT & IVT	Figure S5	PRJEB74106/ SAMEA115526580
50% WT	mix of HEK293 WT & IVT	Figure S5	PRJEB74106/ SAMEA115526581
75% WT	mix of HEK293 WT & IVT	Figure S5	PRJEB74106/ SAMEA115526582
HEK293T-KO-rep1	METTL3 KO	Figure S4	PRJEB40872/ SAMEA7458292
HEK293T-KO-rep2	METTL3 KO	Figure S4	PRJEB40872/ SAMEA7458293
HEK293T-KO-rep2	METTL3 KO	Figure S4	PRJEB40872/ SAMEA7458294
HEK_siMETTL3_input_rep1	METTL3 KD	Figure S4	PRJEB72637/ SAMEA115289934
HEK_siMETTL3_input_rep2	METTL3 KD	Figure S4	PRJEB72637/ SAMEA115289935
HEK_siTRUB1_input_rep1	TRUB1 KD	Figure S4	PRJEB72637/ SAMEA115289938
HEK_siTRUB2_input_rep1	TRUB1 KD	Figure S4	PRJEB72637/ SAMEA115289939

Table 2: Public data sets used in this study.

3 Table S3

sequencing run name	label	Description
Mix_1_A_RTA	unmodified	M6A_M8_S0, M6A_M10_S0, M6A_M14_S0
Mix_2_A_RTA	unmodified	M6A_M11_S0, M6A_M13_S0, M6A_M16_S0
Mix_3_A_RTA	unmodified	M6A_M8_S0, M6A_M10_S0, M6A_M14_S0
Mix_4_A_RTA	unmodified	M6A_M9_S0, M6A_M12_S0, M6A_M17_S0
Mix_5_m6A_RTA	m6A-modified	M6A_M8_S0, M6A_M10_S0, M6A_M14_S0
Mix_6_m6A_RTA	m6A-modified	M6A_M11_S0, M6A_M13_S0, M6A_M16_S0
Mix_7_m6A_RTA	m6A-modified	M6A_M8_S0, M6A_M10_S0, M6A_M14_S0
Mix_8_m6A_RTA	m6A-modified	M6A_M9_S0, M6A_M12_S0, M6A_M17_S0
Mix_9_U_RTA	unmodified	PSU_M1_S0, PSU_M7_S0, PSU_M8_S0
Mix_10_U_RTA	unmodified	PSU_M3_S0, PSU_M5_S0, *PSU_M9_S1*
Mix_11_U_RTA	unmodified	PSU_M0_S0, PSU_M4_S0, PSU_M6_S0
Mix_13_psU_RTA	psU-modified	PSU_M1_S0, PSU_M7_S0, PSU_M8_S0
Mix_14_psU_RTA	psU-modified	PSU_M3_S0, PSU_M5_S0, *PSU_M9_S1*
Mix_15_psU_RTA	psU-modified	PSU_M0_S0, PSU_M4_S0, PSU_M6_S0
Mix_17_A_RTA	unmodified	M6A_M12_S1, M6A_M13_S1, M6A_M17_S0
Mix_18_A_RTA	unmodified	M6A_M13_S0, M6A_M17_S0
Mix_19_A_RTA	unmodified	M6A_M5_S1, M6A_M12_S0
Mix_20_m6A_RTA	m6A-modified	M6A_M12_S1, M6A_M13_S1, M6A_M17_S0
Mix_21_m6A_RTA	m6A-modified	M6A_M13_S0, M6A_M17_S0
Mix_22_m6A_RTA	m6A-modified	M6A_M5_S1, M6A_M12_S0
Mix_23_U_RTA	unmodified	PSU_M2_S1, PSU_M4_S1, PSU_M6_S1
Mix_24_psU_RTA	psU-modified	PSU_M2_S1, PSU_M4_S1, PSU_M6_S1
Mix_51_U_RTA	unmodified	PSU_M12_S0, PSU_M14_S0, PSU_M1_S1
Mix_52_U_RTA	unmodified	PSU_M11_S0, PSU_M13_S0, PSU_M15_S0
Mix_53_U_RTA	unmodified	PSU_M10_S0, PSU_M16_S0, PSU_M4_S2
Mix_54_psU_RTA	psU-modified	PSU_M12_S0, PSU_M14_S0, PSU_M1_S1
Mix_55_psU_RTA	psU-modified	PSU_M11_S0, PSU_M13_S0, PSU_M15_S0
Mix_56_psU_RTA	psU-modified	PSU_M10_S0, PSU_M16_S0, PSU_M4_S2
Mix_57_U_RTA	unmodified	PSU_M13_S0, PSU_M4_S0, PSU_M4_S1
Mix_58_psU_RTA	psU-modified	PSU_M13_S0, PSU_M4_S0, PSU_M4_S1
Mix_59_U_RTA	unmodified	PSU_M2_S0, PSU_M13_S0, PSU_M16_S0
Mix_60_U_RTA	unmodified	PSU_M11_S0, PSU_M2_S1, PSU_M10_S0
Mix_61_psU_RTA	psU-modified	PSU_M2_S0, PSU_M13_S0, PSU_M16_S0
Mix_62_psU_RTA	psU-modified	PSU_M11_S0, PSU_M2_S1, PSU_M10_S0

Table 3: Sequencing runs generated for this study. ENA project ID PRJEB82824.

References

Boulias, K., Toczydłowska-Socha, D., Hawley, B. R., Liberman, N., Takashima, K., Zaccara, S., Guez, T., Vasseur, J.-J., Debart, F., Aravind, L., Jaffrey, S. R., and Greer, E. L. (2019). Identification of the m6Am Methyltransferase PCIF1 Reveals the Location and Functions of m6Am in the Transcriptome. *Molecular Cell*, **75**(3), 631–643.e8.

Chan, A., Naarmann-de Vries, I. S., Scheitl, C. P. M., Höbartner, C., and Dieterich, C. (2024). Detecting m6A at single-molecular resolution via direct RNA sequencing and realistic training data. *Nature Communications*, **15**(1), 3323. Publisher: Nature Publishing Group.

Dai, Q., Zhang, L.-S., Sun, H.-L., Pajdzik, K., Yang, L., Ye, C., Ju, C.-W., Liu, S., Wang, Y., Zheng, Z., Zhang, L., Harada, B. T., Dou, X., Irkliyenko, I., Feng, X., Zhang, W., Pan, T., and He, C. (2023). Quantitative sequencing using BID-seq uncovers abundant pseudouridines in mammalian mRNA at base resolution. *Nature Biotechnology*, **41**(3), 344–354. Publisher: Nature Publishing Group.

Hendra, C., Pratanwanich, P. N., Wan, Y. K., Goh, W. S. S., Thiery, A., and Göke, J. (2022). Detection of m6A from direct RNA sequencing using a multiple instance learning framework. *Nature Methods*, **19**(12), 1590–1598. Number: 12 Publisher: Nature Publishing Group.

Huang, S., Wylder, A. C., and Pan, T. (2024). Simultaneous nanopore profiling of mRNA m6A and pseudouridine reveals translation coordination. *Nature Biotechnology*, pages 1–5. Publisher: Nature Publishing Group.

Koh, C. W. Q., Goh, Y. T., and Goh, W. S. S. (2019). Atlas of quantitative single-base-resolution N6-methyl-adenine methylomes. *Nature Communications*, **10**(1), 5636. Number: 1 Publisher: Nature Publishing Group.

Körtel, N., Rücklé, C., Zhou, Y., Busch, A., Hoch-Kraft, P., Sutandy, F. X. R., Haase, J., Pradhan, M., Musheev, M., Ostareck, D., Ostareck-Lederer, A., Dieterich, C., Hüttelmaier, S., Niehrs, C., Rausch, O., Dominissini, D., König, J., and Zarnack, K. (2021). Deep and accurate detection of m6A RNA modifications using miCLIP2 and m6Aboost machine learning. *Nucleic Acids Research*, **49**(16), e92.

Liu, C., Sun, H., Yi, Y., Shen, W., Li, K., Xiao, Y., Li, F., Li, Y., Hou, Y., Lu, B., Liu, W., Meng, H., Peng, J., Yi, C., and Wang, J. (2023). Absolute quantification of single-base m6A methylation in the mammalian transcriptome using GLORI. *Nature Biotechnology*, **41**(3), 355–366. Number: 3 Publisher: Nature Publishing Group.

Zhang, M., Jiang, Z., Ma, Y., Liu, W., Zhuang, Y., Lu, B., Li, K., Peng, J., and Yi, C. (2023). Quantitative profiling of pseudouridylation landscape in the human transcriptome. *Nature Chemical Biology*, **19**(10), 1185–1195. Publisher: Nature Publishing Group.
